# Supplementary material for: Pneumolysin boosts the neuroinflammatory response to Streptococcus pneumoniae through enhanced endocytosis
Source: Nat Commun. 2022 Aug 26;13:5032. doi: 10.1038/s41467-022-32624-2 (PMC9418233; doi:10.1038/s41467-022-32624-2)
Supplement: Supplementary file 1 — Supplementary Information [file 41467_2022_32624_MOESM1_ESM.pdf]

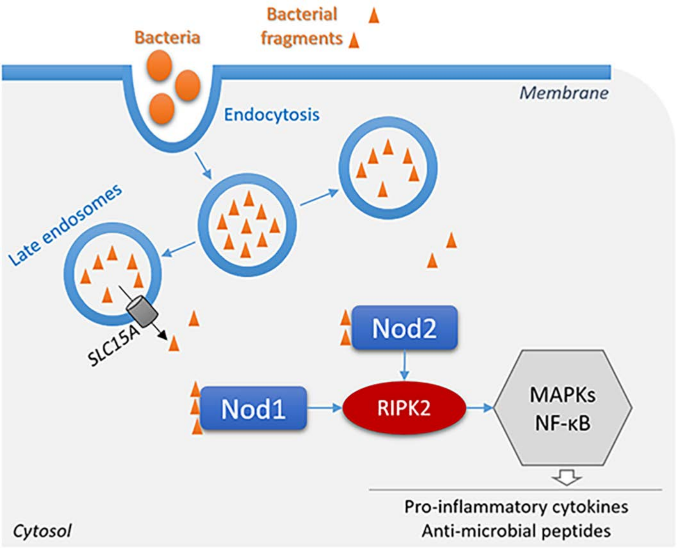

Supplementary Figure S1. Schematic overview of the activation of the Nod receptors by endocytosis of the ligands.

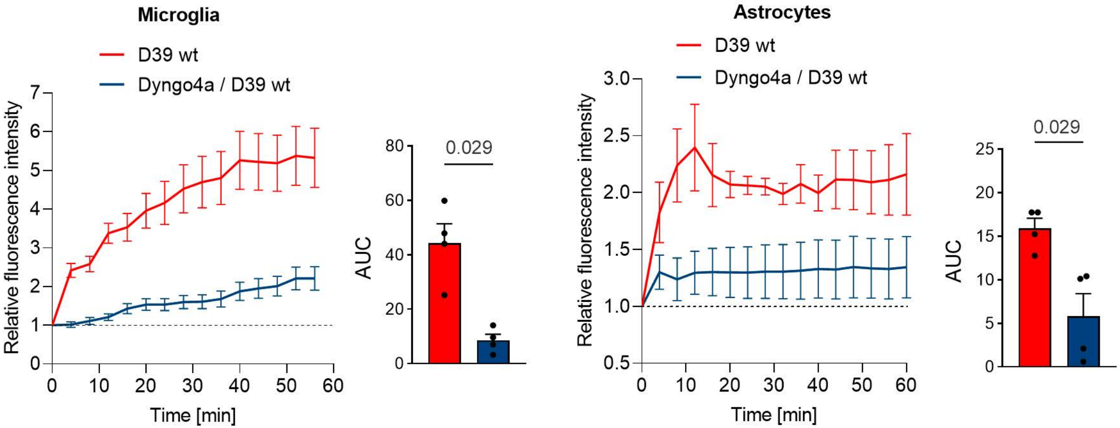

Supplementary figure S2. Dynamin inhibition by 10 µM Dyngo4a diminishes the enhanced FM 4-64 endocytosis following exposure to wild-type D39 lysates in both microglia and astrocytes. All the values represent the mean ± SEM, each dot symbol indicates an independent experiment (n=4 experiments), Mann-Whitney U-test, all tests are two-tailed, exact p-values are indicated (if significant). Source data are provided as a Source Data file.

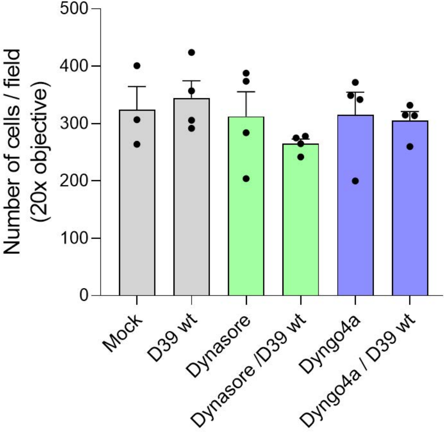

Supplementary Figure S3. The number of DAPI-stained glial cell nuclei with normal morphology remained unchanged 24 h after continous incubation. The applied concentrations were identical to the ones used throughout the work. All values represent the mean ± SEM, each dot symbol indicates an indipendent experimental value (n=4 for all except for mock (n=3)), one-way ANOVA. Source data are provided as a Source Data file.

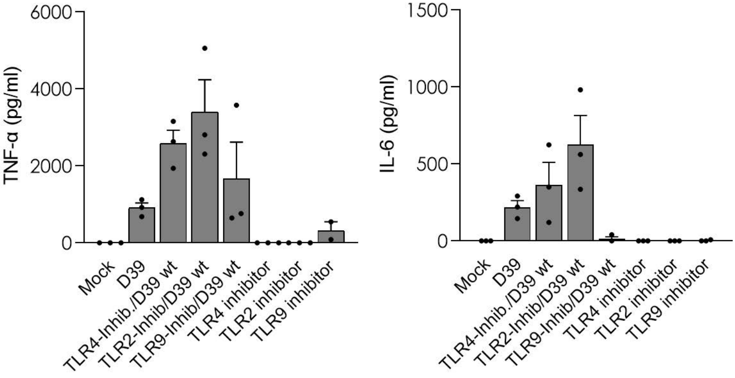

Supplementary figure S4. TNF-α release following challenge with the D39 wild-type lysates is not inhibited by incubation with inhibitors for TLR2 (CU CPT 22, 10 µM), TLR4 (C34, 10 µM) and TLR9 (hydroxychloroquine sulfate, 40 µM) for 36 h. IL-6 is only inhibited by hydroxychloroquine. All the values represent the mean ± SEM, dot symbols indicate the independent experimental values (n=3 independent experiments) Source data are provided as a Source Data file.

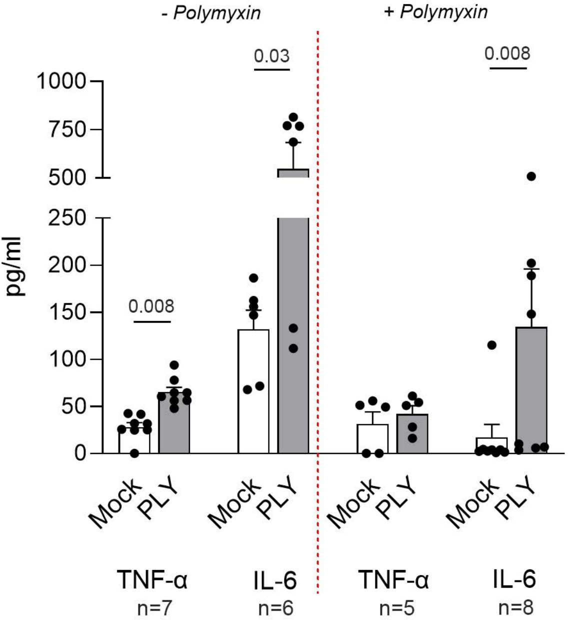

Supplementary figure S5. The elevation in the production of TNF-α and IL-6 in the mixed glial cultures incubated with 4 HU/ml PLY for 24 h is eliminated (TNF-α) or strongly reduced (IL-6) once LPS is removed (see Methods). All values represent the mean ± SEM, Wilcoxon matched pairs test, all test are two-tailed, exact p-values are indicated (if significant). Each symbol represents an independent experiment, n is additionally indicated. Source data are provided as a Source Data file.

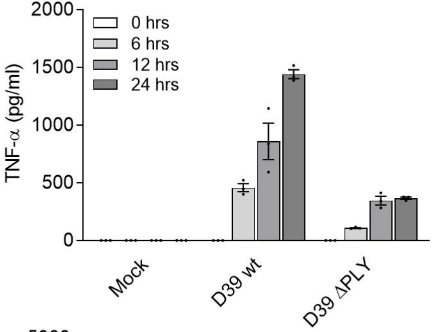

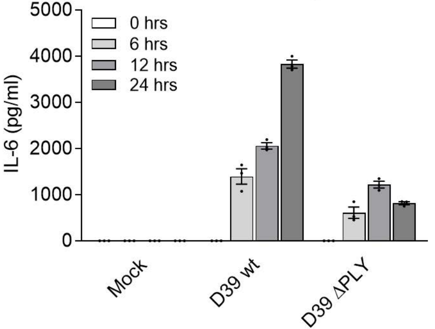

Supplementary Figure S6. Inflammatory cytokine release after incubation with D39 wt lysate or with lysate from PLY-deficient D39 (D39 ΔPLY) ($1x10^7$ CFU/ml) for 0, 6, 12 and 24 h. All values represent the mean ± SEM, dot symbols indicate the independent experimental values, for all groups n=3 independent experiments. Source data are provided as a Source Data file.

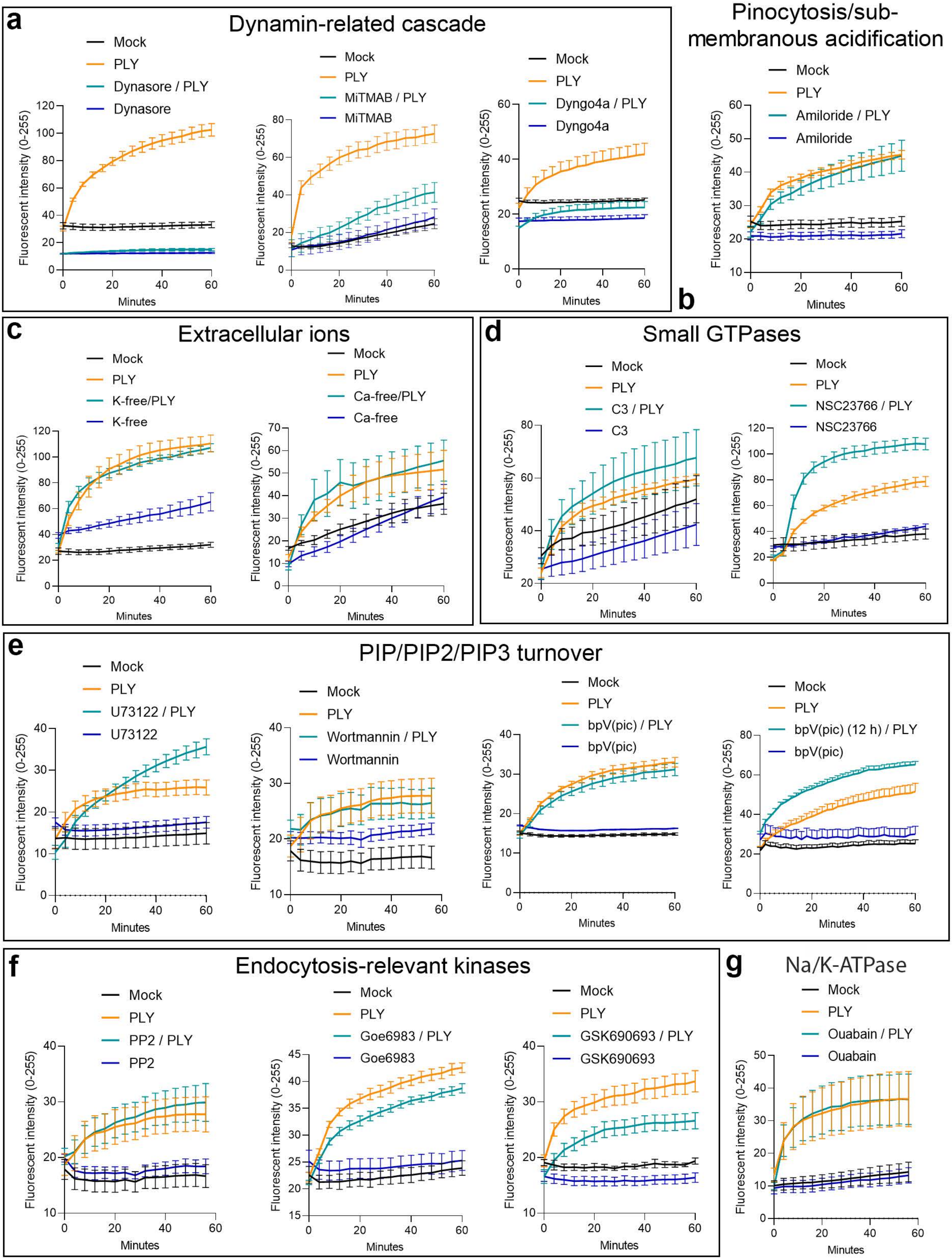

Supplementary figure S7. Raw data (not normalized to mock (vehicle)-treated or inhibitor-treated controls) for all inhibitors/inhibitory conditions on FM4-64 endocytosis after exposure to 2 HU/ml PLY. **a.** Effect of dynamin-specific inhibitors. **b.** Effect of the pinocytosis/sub-membranous acidification inhibitor amilorid. **c.** Effect of extracellular ion depletion. **d.** Effect of the small GTPases RhoA and Rac1. C3 - C3 transferase, RhoA GTPase inhibitor; NSC23766 - Rac1 GTPase inhibitor. **e.** Effect of modulators of the phosphatidylinositol phosphates PIP/PIP2/PIP3. U73122 - phospholipase C inhibitor; Wortmannin - PI3K inhibitor; bpV (pic) - PTEN inhibitor. **f.** Effect of endocytosis-relevant kinases. PP2 - broad-spectrum Src-kinase inhibitor; Goe6983 - broad-spectrum PKC inhibitor; GSK690693 - Akt-kinase and PKCepsilon inhibitor. **g.** Effect of the Na/K-ATPase blocker ouabain. The concentrations are listed in the Materials and methods and in Table 1. All values represent the mean ± SEM. Source data are provided as a Source Data file (included together with the normalized data in the datasheet „Figure 6").

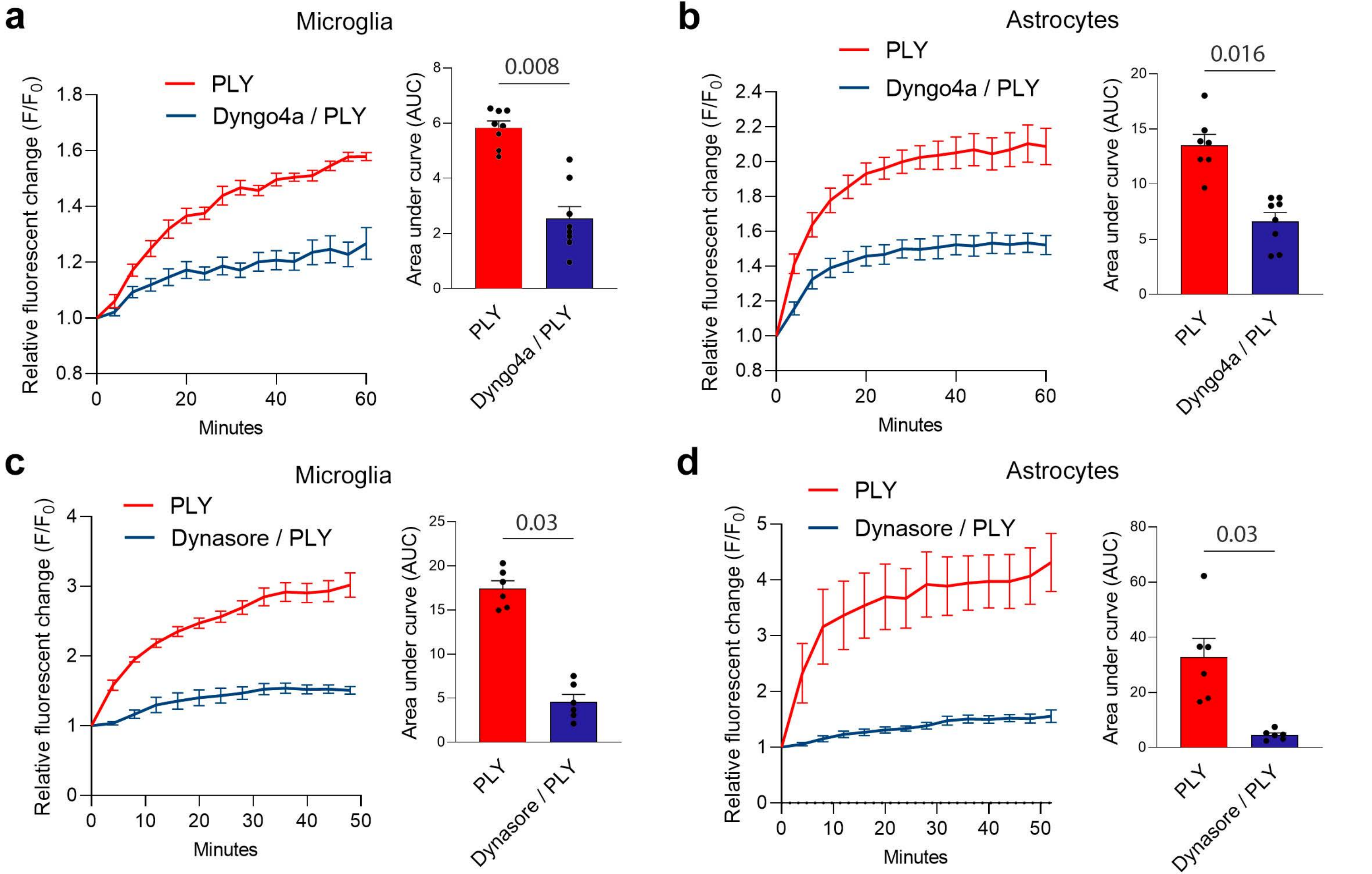

Supplementary Figure S8. Inhibition of endocytosis by 2 HU/ml PLY by 10 µM Dyngo4a (a, b) and Dynasore (c, d) in astrocytes (b, d) and microglia (a, c). All values represent the mean ± SEM, each dot symbol indicates an independent experiment (a. n=8; b. n=7; c. and d. n=6), Wilcoxon matched pairs test, all tests are two-tailed, exact p-values are indicated (if significant). Source data are provided as a Source Data file.

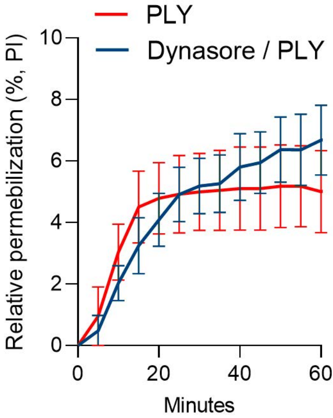

Supplementary Figure S9. Propidium iodide (PI) staining of the chromatin of mixed glia as a sign of acute membrane permeabilization after exposure to 2 HU/ml PLY with and without 10 µM Dynasore treatment. All values represent the mean ± SEM, n=4 independent experiments. Source data are provided as a Source Data file.

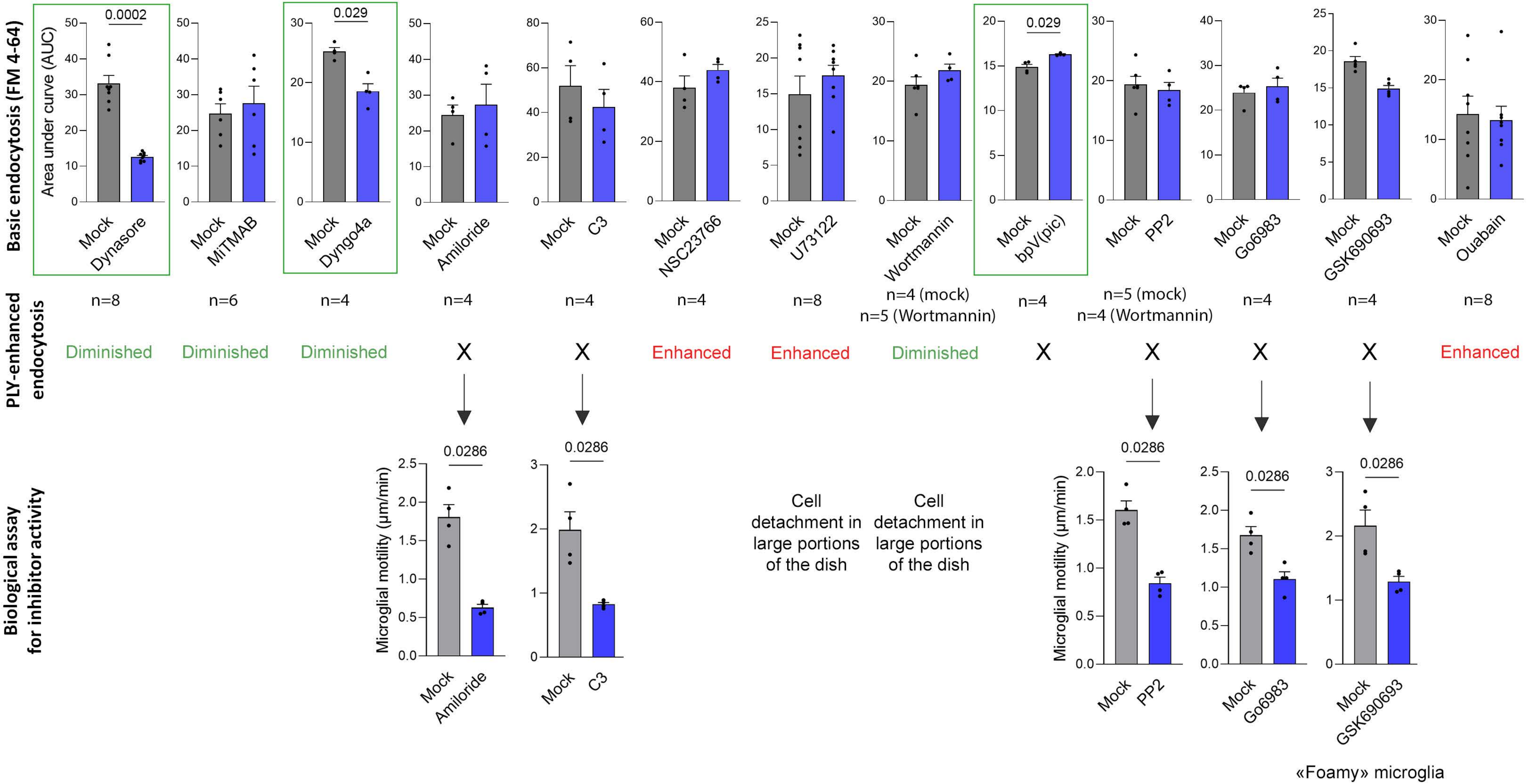

Supplementary Figure S10. Validation of the inhibitor effects. In the first row, the inhibitors that influence normal endocytosis are outlined in green. In the second row, the inhibitors that influence PLY-enhanced endocytosis are indicated. To validate the biological activity of the rest of the inhibitors in our system, we tested their effects on microglia motility, known to be influenced by all of them (for exact references see Material and Methods). All values represent the mean ± SEM, dot symbols indicate the independent experimental values. Exact p-values are presented where significant, Mann-Whitney U-test or Wilcoxon paired test (paired or unpaired data). Source data are provided as a Source Data file.

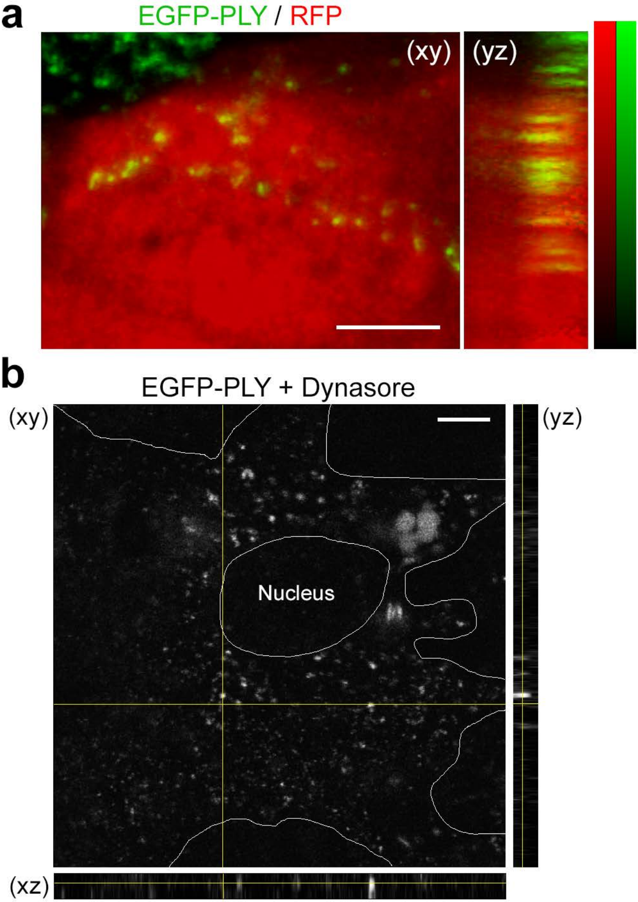

Supplementary figure S11. Dynamin-independent internalization of recombinant GFP-PLY. a. The fluorescently tagged form of PLY (EGFP-PLY) (4 HU/ml, 10 min incubation) is internalized by an astrocyte transfected with mRFP (real-colour presentation) (red and green LUT). b. Pretreatment with Dynasore fails to block EGFP-PLY toxin internalization (as seen in the YZ plane). Scale bar: 5 µm (gray LUT). All experiments were replicated with identical outcome at least 5 times.

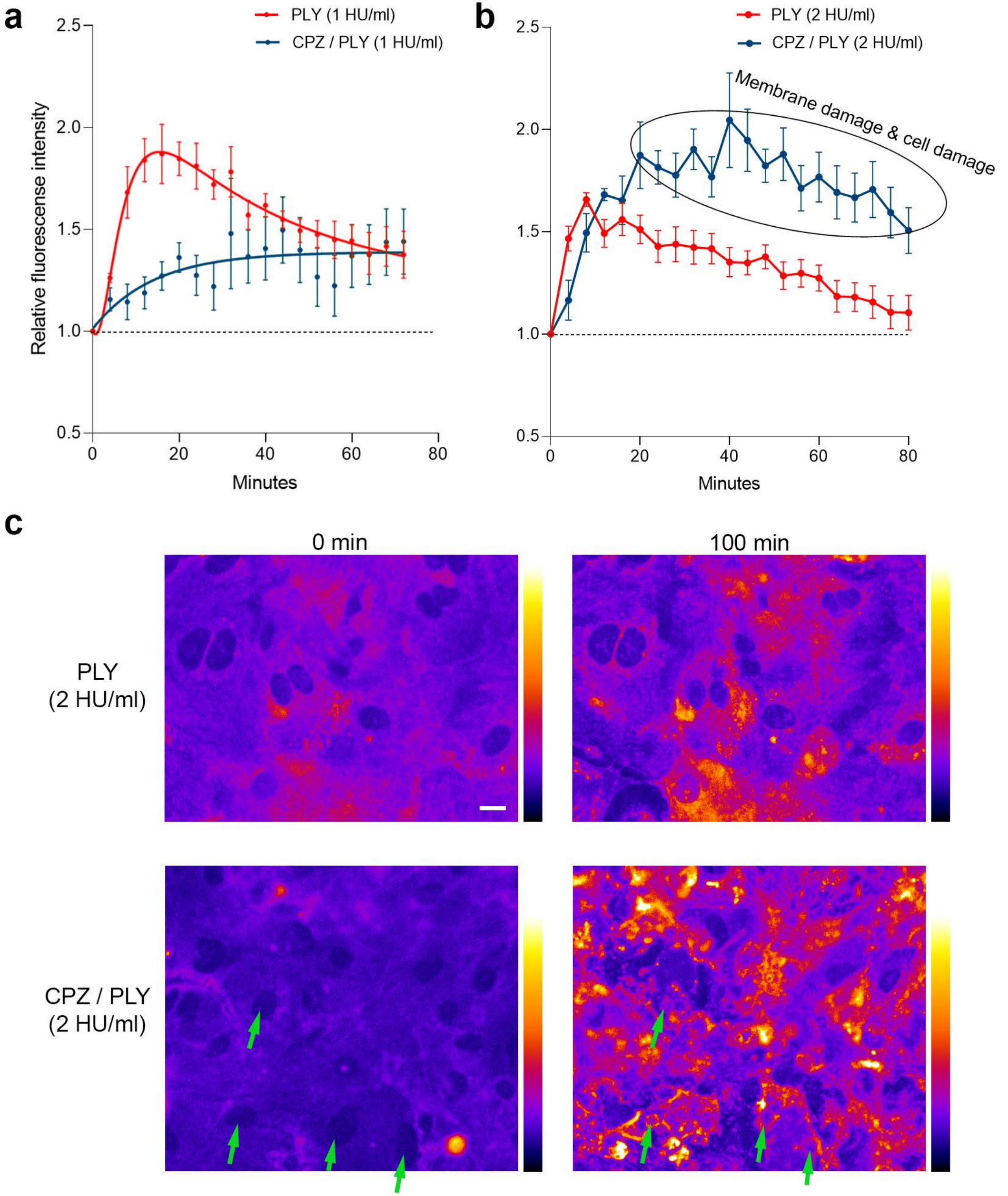

Supplementary figure S12. Endocytosis modulation by 10 mM chlorpromazine (CPZ) in mixed glial culture. A. Inhibition of the PLY-enhanced endocytosis (1 HU/ml toxin and an FM4-64 assay) by chlorpromazine. B. At PLY concentrations of 2 HU/ml and higher, despite mild delay of endocytosis, there was a massive increase of toxicity and membrane blebbing, consistent with enhanced PLY effect. C. Exposure of the cells to PLY alone leads to an enhancement of endocytosis without any sign of cell damage (normal nuclei and membrane configuration). When exposed together with CPZ, the sub-lytic effects of PLY in culture are turned into lytic, leading to nuclear shrinkage (green arrows point to the same nuclei before and after treatment) and massive membrane damage (pseudocolor Fire LUT (ImageJ)). Scale bar: 20 μM. All the values represent the mean± SEM, n=4-5 independent experiments. Source data are provided as a Source Data file.

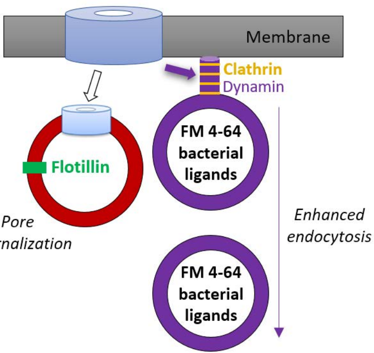

Supplementary figure S13. Schematic diagram of the toxin internalization through flotillin-positive vesicles, and the enhanced endocytosis of classical endosomes through a dynamin-dependent mechanism, which is toxin-dependent too, but these vesicles do not contain the toxin.
