# Supplementary material for: Pneumolysin boosts the neuroinflammatory response to Streptococcus pneumoniae through enhanced endocytosis
Source: Nat Commun. 2022 Aug 26;13:5032. doi: 10.1038/s41467-022-32624-2 (PMC9418233; doi:10.1038/s41467-022-32624-2)
Supplement: Supplementary file 3 — Description of Additional Supplementary Files [file 41467_2022_32624_MOESM3_ESM.pdf]

Description of Additional Supplementary Files

Supplementary Movie 1. Live imaging of FM4-64 endocytosis by D39 lysates in primary glial cells with and without dynasore treatment. LUT color coding Fire (ImageJ) as shown in Figure 2.

Supplementary Movie 2. Live imaging (transmission (left panel) and FM4-64 endocytosis (middle panel)) of primary microglia before and after challenge with 2 HU/ml PLY (treatment indicated). LUT color coding Fire (ImageJ) as shown in Figure 5.

Supplementary Movie 3. 3D reconstruction of endocytosed PLY-EGFP (green) in DsRed-transfected (red) astrocyte. LUT color coding green and red (ImageJ) as shown in Supplementary Figure S11a.
