# Supplementary material for: Pneumolysin boosts the neuroinflammatory response to Streptococcus pneumoniae through enhanced endocytosis
Source: Nat Commun. 2022 Aug 26;13:5032. doi: 10.1038/s41467-022-32624-2 (PMC9418233; doi:10.1038/s41467-022-32624-2)
Supplement: Supplementary file 7 — Reporting Summary [file 41467_2022_32624_MOESM7_ESM.pdf]

nature portfolio

Corresponding author(s): Asparouh ILIEV

Last updated by author(s): August 3, 2022

Reporting Summary

Nature Portfolio wishes to improve the reproducibility of the work that we publish. This form provides structure for consistency and transparency in reporting. For further information on Nature Portfolio policies, see our Editorial Policies and the Editorial Policy Checklist.

Statistics

For all statistical analyses, confirm that the following items are present in the figure legend, table legend, main text, or Methods section.

n/a	Confirmed	
☐	☒	The exact sample size (*n*) for each experimental group/condition, given as a discrete number and unit of measurement
☐	☒	A statement on whether measurements were taken from distinct samples or whether the same sample was measured repeatedly
☐	☒	The statistical test(s) used AND whether they are one- or two-sided<br>*Only common tests should be described solely by name; describe more complex techniques in the Methods section.*
☐	☒	A description of all covariates tested
☐	☒	A description of any assumptions or corrections, such as tests of normality and adjustment for multiple comparisons
☐	☒	A full description of the statistical parameters including central tendency (e.g. means) or other basic estimates (e.g. regression coefficient) AND variation (e.g. standard deviation) or associated estimates of uncertainty (e.g. confidence intervals)
☐	☒	For null hypothesis testing, the test statistic (e.g. *F*, *t*, *r*) with confidence intervals, effect sizes, degrees of freedom and *P* value noted<br>*Give P values as exact values whenever suitable.*
☒	☐	For Bayesian analysis, information on the choice of priors and Markov chain Monte Carlo settings
☒	☐	For hierarchical and complex designs, identification of the appropriate level for tests and full reporting of outcomes
☒	☐	Estimates of effect sizes (e.g. Cohen's *d*, Pearson's *r*), indicating how they were calculated

Our web collection on statistics for biologists contains articles on many of the points above.

Software and code

Policy information about availability of computer code

Data collection	ZEN 2.0 software (Zeiss Microsystems, Jena), IonWizzard version 6.3 (IonOptics Ltd., Dublin, Ireland), Olympus Cell ^M 3 (Build 1243) imaging package (Olympus Deutschland GmbH, Hamburg, Germany), Gen5 package (version 2.00.18, BioTek Instruments, Inc., Winooski, VT, USA)
Data analysis	Image J (NIH, Bethesda, USA), FIJI (Johannes Schindelin and team), GraphPad Prism (9.4.1., GraphPad Software Inc., La Jolla, CA, USA), ZEN 2.0 (Zeiss Microsystems, Jena, Germany), IonWizzard (IonOptics Ltd., Dublin, Ireland)

For manuscripts utilizing custom algorithms or software that are central to the research but not yet described in published literature, software must be made available to editors and reviewers. We strongly encourage code deposition in a community repository (e.g. GitHub). See the Nature Portfolio guidelines for submitting code & software for further information.

Data

Policy information about availability of data

All manuscripts must include a data availability statement. This statement should provide the following information, where applicable:

- Accession codes, unique identifiers, or web links for publicly available datasets
- A description of any restrictions on data availability
- For clinical datasets or third party data, please ensure that the statement adheres to our policy

All data generated or analyzed during this study are included in this published article (and its supplementary information files) and are available as datasets in the repository https://doi.org/10.48620/63. Confocal, fluorescent live imaging and electron microscopy raw data is available from the corresponding author on reasonable request (due to very large size).

Field-specific reporting

Please select the one below that is the best fit for your research. If you are not sure, read the appropriate sections before making your selection.

☒ Life sciences ☐ Behavioural & social sciences ☐ Ecological, evolutionary & environmental sciences

For a reference copy of the document with all sections, see nature.com/documents/nr-reporting-summary-flat.pdf

Life sciences study design

All studies must disclose on these points even when the disclosure is negative.

Sample size	For the animal experiments, sample size was determined using G-power software. For all other in vitro test - at least 4 replicates, followed by G-power software analysis of the feasibility of additional replicates (not more than 12) to identify a possible trending difference.
Data exclusions	No data was exlcuded.
Replication	All in vitro experiments were replicated successfully in triplicate or more (indicated as n in all tests).
Randomization	The animals were randomized into two groups containing equal numbers of male and female animals, as well as randomized between cages.
Blinding	At all steps of the animal experiments and the subsequent analysis, the animals and the corresponding samples were blinded (where possible; clinical picture chnages after chlorpromazine treatment in the first hours, providing phenotypic evidence for treatment). For all in vitro experiments, blinding was applied during scan through an automatic scan of all treatment/control groups using predefined scan positions. Unblinding followed after scan and before automatic analysis of the data.

Reporting for specific materials, systems and methods

We require information from authors about some types of materials, experimental systems and methods used in many studies. Here, indicate whether each material, system or method listed is relevant to your study. If you are not sure if a list item applies to your research, read the appropriate section before selecting a response.

Materials & experimental systems

n/a	Involved in the study
☐	☒ Antibodies
☐	☒ Eukaryotic cell lines
☒	☐ Palaeontology and archaeology
☐	☒ Animals and other organisms
☒	☐ Human research participants
☒	☐ Clinical data
☒	☐ Dual use research of concern

Methods

n/a	Involved in the study
☒	☐ ChIP-seq
☒	☐ Flow cytometry
☒	☐ MRI-based neuroimaging

Antibodies

Antibodies used	Anti-flotillin 1 rabbit antibody (ab41927, Abcam), anti-Arf6 rabbit antibody (ab77581, Abcam), goat anti-rabbit Cy3 (111-166-144, Jackson)
Validation	Anti-flotillin was validated in flotillin-1 ko cell line by the manufacturer (https://www.abcam.com/flotillin-1-antibody-ab41927.html)<br>anti-Arf6 was validated in ARF6 knockout HAP1 cell lysate by the manufacturer (https://www.abcam.com/arf6-antibody-ab77581.html)<br>In all antibody experiments, we performed primary antibody isotype controls.

Eukaryotic cell lines

Policy information about cell lines

Cell line source(s)	HEK293 cell line, DSMZ (Braunschweig, Germany)
Authentication	The line was purchased and used according to the provider. It was not further authenticated.
Mycoplasma contamination	Tested negative by PCR and DAPI staining.
Commonly misidentified lines (See ICLAC register)	Not used.

Animals and other organisms

Policy information about studies involving animals; ARRIVE guidelines recommended for reporting animal research

Laboratory animals	C57BL6JRj, postnatal day 3-5 for culture preparation, 8-12 week old for animal experiments, equal ratio 1:1 male:female. Provider - Janvier.<br>Animals were housed in 12/12 h light/dark cycle 21 ± 2 ^{o}C, 56% relative humidity with lights turned on at 08:00 and turned off at 20:00

Wild animals	Not used
Field-collected samples	Not used.
Ethics oversight	Animal Protection Commission of the Canton Bern under No. BE103/2020 and full compliance with local legislation.
